# Improving Efficiency in Dental School Clinics by Computerizing a Manual Task

**DOI:** 10.3390/dj7020044

**Published:** 2019-05-01

**Authors:** Vladyslav Virun, Gurmukh Singhe Dhaliwal, Chieh-Han (Jeffrey) Liu, Pranshu Sharma, Harleen Kaur, Romesh P. Nalliah

**Affiliations:** School of Dentistry, University of Michigan, Ann Arbor, MI 48109, USA; romeshn@umich.edu (V.V.); gurridhaliwal@gmail.com (G.S.D.); jeffreyliu80330@gmail.com (C.-H.J.L.); pranshu.sh@gmail.com (P.S.); romesh.aus@gmail.com (H.K.)

**Keywords:** efficiency, patient experience, privacy

## Abstract

*Background:* We computerized a formerly manual task of requesting dental faculty to conduct quality checks on student providers during patient encounters. We surveyed student providers who experienced the manual and computerized versions of the faculty request process for one year each. *Methods:* All surveys were emailed to student providers and there were no reminders or incentives to complete the survey. Simple descriptive data were used to present the results of the study and Institutional Review Board (IRB) approval was provided by the University of Michigan Medical School Committee on Human Research (HUM00131029) on 1 June 2018 *Results:* The response rate for the survey was 47.1%. A total of 16.1% of student providers reported that the Faculty Request System (FRS) helped them save 1–10 min per clinic session, 22.3% said it saved them 11–20 min, 29.5% said it saved them 21–30 min, 21.4% said it saved 31–40 min, 2.67% said it saved 41–50 min, and 7.14% said it saved more than 50 min per clinic session. Regarding how student providers used the additional time they gained from the FRS, 96.4% said they used some of the time to write up their notes, 88.4% said they used some of the time to discuss treatments with their patients, 83.9% said they engaged in general conversation with their patients, 81.3% said they took care of other patient-related duties, while 1.8% said they had less time available after the implementation of the FRS. *Conclusions:* The FRS enabled student providers to remain with their patients for almost a full 30 min more (during a 3 h session). This paper describes several benefits experienced by student providers, and the resulting impacts on patient experiences.

## 1. Introduction

The University of Michigan School of Dentistry (UMSOD) pre-doctoral clinics manage about 100,000 patient encounters every year. In dental school clinics (DSCs) in the United States (US), care is delivered by dental student providers under the close supervision of licensed dental professionals. Each step of the clinical process is checked for quality before the student provider can proceed to the next step. UMSOD has very strong patient satisfaction scores, and surveys indicate that 89.6% of our patients would “very likely recommend” our services to family and friends and a further 8.3% said they would “somewhat likely recommend” our services. However, some patients raised concerns that too much time is spent “waiting for faculty to check steps” during appointments. Most DSCs in the US suffer shortages of faculty members [1], so any improvements in efficiency have the potential to have nationwide benefit to other DSCs.

We conducted evaluations of our clinic operations and noted that, on average, student providers spent about 42 min per three-hour session waiting for faculty to come to operatory rooms and check their work [2]. This was worsened by the fact that UMSOD student providers called faculty by waiting in a physical line as faculty visited each student and patient, known as the “Pied Piper” effect. Attempts were made previously to use a signup sheet, but multiple efforts remained unsuccessful and unsustainable. Therefore, a completely new method was necessary. Additionally, we recognized that this system was a threat to patient privacy as a line of students waited at the foot of the dental operatory while the faculty checked the patient whose student was first in line. Then, the entire group would move to the next patient and so on and so forth. We wanted to improve the patient experience and make the faculty request process more efficient.

UMSOD has an extremely talented information technology team and, under the leadership of the Director for Pre-Doctoral Clinical Education, the Dean of Patient Services, and the Dean for Faculty Affairs and Institutional Effectiveness, they set about building an electronic system to replace the Pied Piper effect—the Faculty Request System (FRS).

Nineteenth century economist John Maynard Keynes wrote an essay in 1930 entitled “Economic Possibilities for our Grandchildren” [3]. In this essay, Keynes described how technology is advancing faster than our ability to retrain and absorb those employees into new tasks, predicting widespread unemployment as a consequence. However, technological advances do not always result in unemployment, as the introduction of new technology can impact a job in one of two ways. The technology may complement the worker and enhance their ability to perform certain tasks, or the technology may replace the worker by automating some or all of their tasks [4]. Clearly, the FRS cannot replace dental faculty and can only complement their work. However, the introduction of this technology was met with significant concern and resistance from faculty and students. The importance of good leadership in navigating this resistance cannot be overstated, as the Pied Piper effect had been the accepted operational method for 30 years.

The goal of the FRS was to automate the Pied Piper process and allow student providers to remain with their patients. Every request for faculty is time-stamped and these business intelligence data are valuable in evaluating and planning staffing. Office workers spend a lot of time in non-value-added activities. While no such data are available on how often this occurs in the dental profession, a 2014 Forbes article suggests that 89% of workers waste at least some time each day [5]. Many improvements to operations were implemented from the use of business intelligence data gathered by the FRS [2]. The focus of this paper is on assessing the impacts of computerizing the process of requesting faculty and to evaluate its effects on student providers and their patients.

On 25 January 2016, the FRS was implemented at UMSOD. This paper is a summary of survey findings on the experiences of all student providers who delivered care in the year prior and the year following the implementation of the FRS. To our knowledge, this is the first publication about a technology implementation in a DSC aimed at improving processes for student providers and their patients.

## 2. Methods

Twelve months after implementing the FRS, we surveyed all student providers who had experienced the old way of requesting faculty (i.e., the Pied Piper effect) and the new way using the FRS (i.e., third- and fourth-year students). Participation in the survey was voluntary and there were no financial incentives to participate. Survey participation reminders were not sent. A Qualtrics survey link was provided by email to all students from the two affected classes—class of 2017 and class of 2018. Survey questions were multiple choice with some opportunity for free-text comments at the end. Participation in the FRS was also voluntary, however, we strongly encouraged students to participate because we realized that faculty could not cope in an environment where some students used the FRS and some did not. IRB approval was gained from the University of Michigan Medical School Committee on Human Research (HUM00131029).

## 3. Results

The response rate for the survey was 47.1% (i.e., 112 individuals overall, or 47.1% of third- and fourth-year dental students). Overall, 86.6% of providers reported that the FRS had improved their learning experience in the clinics. Furthermore, 92.0% reported that the FRS has enabled them to be more efficient in the clinics and 83.0% said that the FRS had enabled them to deliver more services to their patients in a given amount of time.

**Figure 1:**
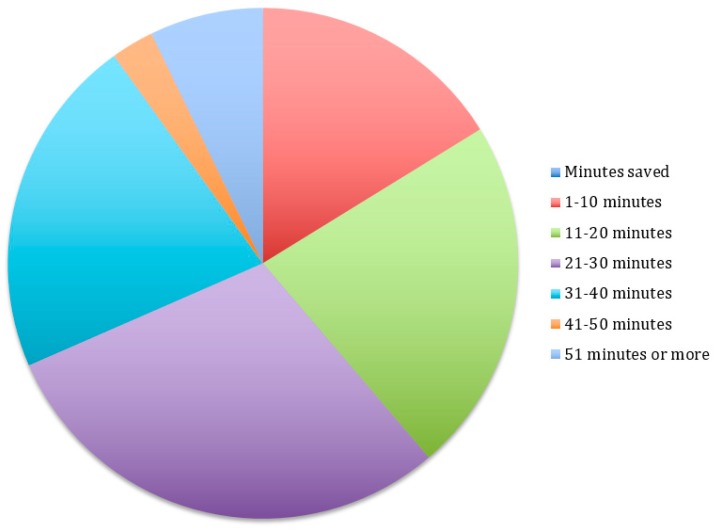
Student reports of the number of minutes saved in every 3 h appointment after implementation of the Faculty Request System (FRS).

Figure 1 demonstrates that 16.1% of student providers reported that the FRS helped them save 1–10 min per clinic session, 22.3% said it saved them 11–20 min, 29.5% said it saved them 21–30 min, 21.4% said it saved 31–40 min, 2.67% said it saved 41–50 min, and 7.14% said it saved more than 50 min per clinic session.

**Figure 2:**
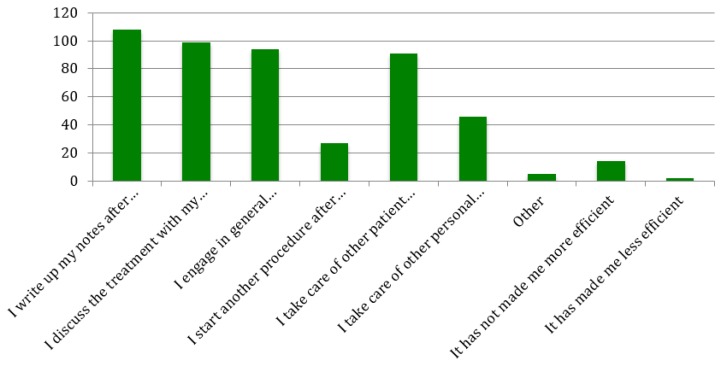
Most frequently reported ways that providers spend extra time gained from the implementation of the FRS (could choose more than one option).

The ways in which student providers used the additional time they gained from using the FRS (Figure 2) were also assessed. Of the respondents, 96.4% said they used some of this time to write up their notes, 88.4% said they used some of the time to discuss treatments with their patients, 83.9% said they engaged in general conversation with their patients, 24.1% said they started a second procedure for patients, 81.3% said they took care of other patient-related duties, 41.1% said they took care of other personal duties, while 1.8% said they had less time available after the implementation of the FRS.

**Figure 3:**
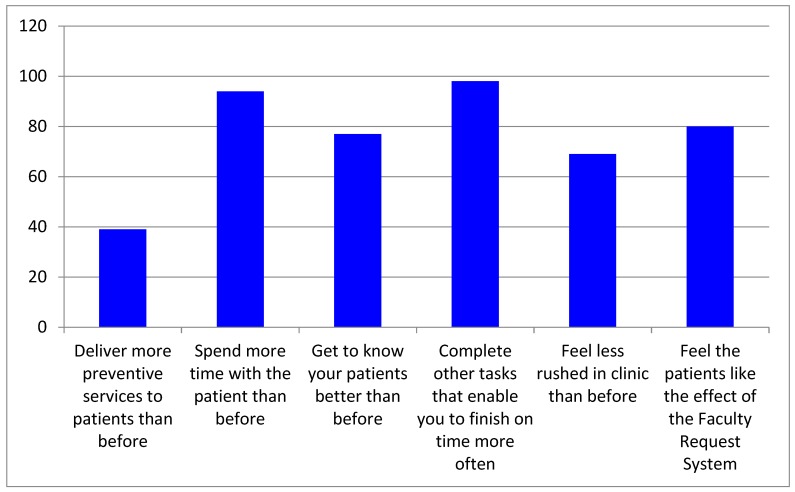
Provider reports of the benefit of implementation of the FRS.

The survey also assessed the benefits that student providers reported compared to the system in place prior to the FRS (see Figure 3). Of the respondents, 34.8% said they delivered more preventive services to their patients, 68.8% said they got to know their patient better through casual conversation, 87.5% said they used the additional time to complete other tasks in order to finish their patient appointment on time more often and 69.4% reported feeling less rushed in the clinics.

## 4. Discussion

The FRS was a tool that was built primarily to improve the patient experience by reducing the need for student providers to leave the dental operatory and leave patients alone. Although we could not reduce the wait time for faculty, we were able to build an electronic process for requesting faculty which would enable the student to remain engaged with the patient. We did not instruct students on how to spend the additional time gained from not having to stand in “Pied Piper” lines, and allowed this to evolve organically. We measured the amount of time that the patient was left alone before and after the implementation of the FRS [2]. We found that the average time moved from 41 min and 36 s to 11 min and 40 s (a savings of almost 30 min). The purpose of the current survey was to understand how students were choosing to use this additional time and report any benefits to the patient.

### 4.1. Productivity

John A. Young reports that 80% of productivity growth in the US is attributable to technology implementation [6]. Although the FRS was not imagined for the purpose of improving productivity, 88.3% of student providers reported that the FRS enabled them to be more efficient in the clinics. Furthermore, 83.8% of survey respondents said that the FRS had enabled them to deliver more services to their patients in a given amount of time. Additionally, when asked how they spent the additional time, 24.3% of student providers reported that they started an additional dental procedure. All of these outcomes suggest an increase in productivity. We reviewed productivity data on the period prior to the implementation of the FRS and found that each appointment in the DSC was associated with an average of 2.6 procedures per session. After the FRS implementation, this number rose slightly to 2.8 and our revenues increased by 12.0% (however, UMSOD is a complex organization and this improvement may not all be attributed to the FRS).

The survey also assessed what student providers are doing more of during the additional time they spend with the patient. Of the survey respondents, 35.1% said they delivered more preventive services. Although we did not find a change in the number of preventive services per patient, a review of 50 charts suggested that several additional preventive services (e.g., fluoride therapy or oral hygiene instruction) were delivered but were not billed.

### 4.2. Efficiency

Research shows that high levels of stress are associated with medical error [7]. Time pressures have been reported as one of the major sources of stress in the dental profession [8]. However, our study showed that 62.1% of student providers reported feeling less rushed in the clinics after implementation of the FRS. A total of 97.3% reported that they used some of the additional time to write up their notes, and 82.0% said they took care of other patient-related duties. As Figure 1 shows, a total of 29.7% said it saved them 21–30 min, 21.6% said it saved 31–40 min and 7.2% said it saved more than 50 min per clinic session. Additionally, a total of 88.3% of student providers said they used the time gained by using the FRS to complete other tasks so that they were more likely to finish on time more often.

The FRS enabled us to know when appointments were finishing and when they were starting. Prior to the implementation of the FRS we did not have this data. Therefore, we reviewed the data of appointment finish times since the implementation of the FRS and found that fewer student providers were finishing late. In February 2016 (soon after implementation), we found that 6.8% of appointments finished after the scheduled end times for the clinic. However, in February 2017, we found that only 4.9% finished late. This small improvement meant that approximately 432 additional appointments finished on time in 2017 compared to 2016. In February 2018 we found that 1.0% finished late, meaning that 634 additional appointments finished on time in 2018 compared to 2016. Therefore, the overall impact on those 634 patients has the potential to be very valuable.

### 4.3. Accountability

The only goal of the FRS was to automate the Pied Piper process in order to allow student providers to remain with their patients. However, every request for a faculty is time-stamped and this business intelligence data could be valuable in evaluating and planning staffing for DSCs. We found that once a clinic session starts, faculty have about 4.0% downtime (time where there is no active request for them) which translates to about 7 min per 3 h session. In fact, we found that 83.0% of the time (close to 2.5 hours in a 3 h session) there was more than one request for faculty (which means they are overloaded). Additionally, identifying slower periods enabled us to make changes to the clinic sessions by staggering start times. This took advantage of the “lull” period by using this time for student requests, meaning busier times were reduced.

## 5. Patient Experience

A total of 89.2% of student providers said that they used some of the time gained from using the FRS to discuss and explain treatment with their patients. Research from primary care medicine shows that time spent by the physician in health education improved patient satisfaction in addition to the effects of treatment [9]. It is also notable that 84.7% of student providers reported that they used some of the additional time to engage in general conversation with their patient and 69.4% said they got to know their patient better than before the FRS implementation.

## 6. Conclusions

The Faculty Request System is the computerization of a manual queuing mechanism. This system enabled student providers to remain with their patients for almost a full 30 min more (during a 3 h session) than before implementation of the FRS. This paper described several benefits experienced by student providers, as well as how the FRS has impacted the patient experience.
